# Barriers and Enablers to Shared Decision Making in Psychiatric Medication Management: A Qualitative Investigation of Clinician and Service Users' Views

**DOI:** 10.3389/fpsyt.2021.678005

**Published:** 2021-06-17

**Authors:** Emma Kaminskiy, Yaara Zisman-Ilani, Nicola Morant, Shulamit Ramon

**Affiliations:** ^1^School of Psychology and Sports Science, Anglia Ruskin University, Cambridge, United Kingdom; ^2^Department of Social and Behavioral Sciences, College of Public Health, Temple University, Philadelphia, PA, United States; ^3^Department of Clinical, Educational and Health Psychology, Division of Psychology and Language Sciences, University College London, London, United Kingdom; ^4^Division of Psychiatry, Faculty of Brain Sciences, University College London, London, United Kingdom; ^5^School of Health and Social Work, University of Hertfordshire, Hatfield, United Kingdom

**Keywords:** shared decision making, barriers, facilitators, co-production, medication, psychiatry, coercion, stigma

## Abstract

Shared decisionmaking (SDM) is a recommended health communication approach in mental health settings. Yet, implementation of SDM in psychiatric consultations discussing medication management is challenging. Insufficient attention has been given to examine the views of both clinicians and service users together about the experiences of SDM in psychiatric medication management. The purpose of this paper is to examine the views of service users, community psychiatric nurses, and psychiatrists about enablers and barriers of SDM. A thematic analysis of 30 semi structured interviews with service users, psychiatrists, and community psychiatric nurses, in a community mental health team in the UK, was conducted. A service user advisory group was involved in all phases of the research cycle, including data collection, analysis, and dissemination. The results offer a detailed contextualized account of how medication decisions are made. For psychiatrists and service user participants SDM is seen as a way of enhancing service users' engagement in and control over treatment decisions. While psychiatrists value the transactional benefits of SDM, service user participants and psychiatric nurses conceptualize SDM as a long-term endeavor embedded within therapeutic partnerships. For service users these partnerships mitigate acknowledged problems of feeling unable to be fully involved during times of crisis. This study identified a range of barriers and facilitators to SDM concerning psychiatric medications from the lived experience of service users and the professional experience of clinicians. Furthermore, it indicates new potential intervention points to support SDM in psychiatric medication decisions.

## Introduction

Psychiatric medications are often considered to be the cornerstone of psychiatric care (1, 2). Yet, many mental health service users do not choose to take medication consistently or at all (up to 75%) despite the increased risk of relapse (1, 3–5). Overall, inconsistent use of psychiatric medications may be a reflection of ineffective or lack of communication between psychiatrists and their patients regarding the harms and benefits of the medications, the range of options and varying side effects, and how psychiatric medications can facilitate recovery (6). Inconsistent use of psychiatric medications is associated with mental health services disengagement, frequent emergency department visits, hospitalizations, and more-severe symptoms over time (3, 7).

Shared decision making (SDM) is a recommended healthcare communication practice, with the potential to improve treatment decisions and health outcomes (8, 9). In an SDM process the emphasis is on the patient as a person, taking into account patient's preferences, needs, beliefs, and concerns about etiology and treatment, and incorporating patients' experiential knowledge. SDM can also promote the person's involvement in their care, services engagement and treatment adherence (8, 10). In mental health care, SDM has been also associated with to promotion of self-determination, self-directed care, and the personal recovery approach, dominant across most health care systems in the Europe and Anglophone countries (11, 12). SDM is viewed as an ethical imperative across mental health systems globally (13). In the UK, SDM has been embedded in policy and practice guidelines for the last two decades and forms part of statutory requirements and training among mental health practitioners (14). SDM can lead to reduction in stigma and increased involvement (15) and recovery outcomes, such as improved quality of life and symptom severity (16) and patient autonomy (17).

Although the promise of SDM in mental health has discussed widely the evidence base remains weak and cultural barriers to implementation appear paramount (18–20). Service users often express desire to be involved in decisions and prefer SDM to other models of patient participation (11, 21–23), yet they often report lack of sufficient antipsychotics decision-making involvement and knowledge about antipsychotics risks and benefits (6, 24). Often concerns are expressed about the competence of service users to be involved due to issues of decisional capacity and insight, along with common misconceptions that is it already happening, and that not all patients want SDM (25–27).

Research into how SDM happens in meetings for psychiatric medication management have found that psychiatrist often employ persuasion in encounters with service users, and concerns about adverse effects are often ignored (28, 29). Clinicians, especially psychiatric care providers, often struggle with using SDM in psychiatric medication decisions as SDM is often perceived to involve risk for clinicians, such as liability or making clinical errors (30). Yet, little research exists about cultural and structural enablers and barriers in these settings. In addition, the views of practitioners and service users taken together are rarely reported yet offer important insights into areas of divergence in views (18).

The purpose of the present qualitative study is to address these knowledge gaps and explore cultural and structural barriers and enables of SDM in psychiatric medication management from the point of view of services users and clinicians in a UK community mental health setting.

## Method

### Setting and Recruitment

Participants were recruited from a community mental health team (CMHT) that provided care to people with serious mental illness (SMI) in the Eastern region of England, UK. Recruitment was conducted *via* key workers in the CMHT and leaflets were distributed in the local outpatient clinic.

### Ethics

Ethical permission was obtained from an NHS research ethics committee (#10-H0311-58) and all participants gave full informed written consent. A broad inclusion criteria was used to include all psychiatrists, mental health nursing staff, and adult service users receiving services from the CMHT at the time of study. People with a lack of capacity to give informed consent and inadequate knowledge of English language were excluded and diagnosis was not considered in the inclusion criteria. Service user participants received £10 GBP as a token of thanks and to reimburse for any travel expenses accrued. Staff did not receive any reimbursement for participation.

### Interviews and Data Collection: A Co-Produced Effort

A project advisory group [consisting of three mental health service users, a community psychiatric nurse (CPN), and one carer] contributed to the development of the interview guide, study design, data collection and analysis. The interview guides for both practitioner and service user interview schedules comprised of three sections: (1) general background questions relevant to the topic (e.g., details of content of recent meetings) (2) participants' views and experiences of involving service users in decision making about psychiatric medication (3) discussion of recent memorable successful and unsuccessful meetings where medication was reviewed (e.g., “what was it about this meeting that particularly stood out for you as being successful/unsuccessful?”). This style of questioning is known to assist accessing more subtle views, less likely to be retrieved along a more general line of questioning. Interviews lasted between 45 and 75 min. Service user interviews were undertaken by the lead researcher (EK), alongside co-researchers (service user members of the advisory group). Clinician interviews were undertaken by a sole interviewer (EK). All interviews were audio recorded and transcribed using standard conventions, and subsequently imported into the qualitative analysis software tool, N Vivo (Version 10).

### A Collaborative Data Analysis

We used an inductively oriented thematic analysis aiming for a rich description of the entire data set and focusing on meaning and lived experience of the phenomenon (31). Including co-researchers in analysis supported this goal and offered particular benefits (32). Best practice guidelines were conformed to ensuring a transparent and deliberate process for coproduced knowledge (32, 33).

A collaborative two stage approach was adopted. During stage one the first author coded all interview transcripts and a sub sample were coded independently by the third author and additional team member, followed by group discussion where themes were continually and iteratively reviewed.

Stage two involved a co-production, collaborative analysis of several transcripts from the service user interview data, undertaken with all members of the project advisory group. During this phase six group analysis meetings took place. These meetings involved group coding of transcripts, discussions of theme structure, and salience and divergence.

## Results

Our sample included 30 participants, of which 15 were service users, 7 (out of a total of 8) psychiatrists working in the pathway, and 8 (out of a total of 9) nurses. Background information for participants are shown in Tables 1, 2. Service users aged from 22 to 54 (*M* = 36, SD = 10.18) and 60% (*N* = 9) were women. The CMHT served ~260 service users, with diverse needs and SMI. The maximum length of stay within this service was 18 months. The majority of service user participants in this study were prescribed antidepressants (*n* = 14, 93.3%), followed by antipsychotics (*n* = 4, 26.6%), mood stabilizers (*n* = 2, 13.3%) and sleep medication (*n* = 1, 6.6%). The majority of psychiatrists and all the nurses interviewed had worked in the CMHT for over 6 months.

**Table 1:** Service user participant characteristics.

**Participant pseudonym**	**Age**	**Gender**	**Class/type of medication currently prescribed**	**Named medication, if known**	**Length of time taking psychiatric medication or since medication changed**	**Discusses medication with**
Natasha	31	F	Antidepressant	Unknown	Six months taking current medication	CPN, GP
Holly	36	F	Antidepressant	Trepidone, 200 mg Efexor (or known as Venlafaxine), 300 mg. Also Gabapentin for pain	Taken psychiatric medication on and off since 19 years old. (17 years approx)	Clinical psychologist, GP
			Mood stabilizer (for pain)			
Carrie	38	F	Antidepressant	Fluoxetine, Mirtazapine, tryptophan, Aripiprazole	Taken psychiatric medication on and off for around 10 years. Most recent change 4 months ago	Psychiatrist, GP, social worker
			Antipsychotic			
Carl	28	M	Antidepressant	Unknown	Just over a year	GP, CPN, psychiatrist
Noel	47	M	Antipsychotics	Haloperidol, amitriptyline, resperidone, depixol	On and off for 30 years.	Psychiatrist, CPN, social worker
			Antidepressant			
Ziggy	34	F	Antidepressant	Amitriptyline	2 months since new medication	CPN, psychiatrist, GP
Linda	22	F	Antidepressant	Venlafaxine	2 months since new medication	Psychiatrist, GP, CPN
Terry	31	M	Atypical antipsychotic	Closapine 400 mg	2 years. Reduction in dose 9 months ago	CPN, psychiatrist
David	23	M	Unknown	Unknown	4–5 months	Psychiatrist, CPN
Lara	42	F	Antidepressant	Venlafaxine	2 weeks since new medication	GP, psychiatrist
Peter	50	M	Unknown	Unknown	Unknown	GP
Andrew	49	M	Antidepressant	Unknown	Since 2010	Psychiatrist, GP
Lizzy	54	F	Antidepressant	Venlafaxine, lithium (1,000 mg)	Venlafaxine: unknown. Lithium-6 years since dose changed. Total 10-15 years been taking lithium	CPN, psychiatrist
			Mood stabilizer			
Casey	28	F	Antidepressant	Fluoxetine, (60 milligrams) merotratine (200 milligrams), sleeping tablet	Has been taking psychiatric medication for ~2 and a half years	GP, psychiatrist, nurse
			Sleeping tablets			
Rosie	24	F	Antidepressant	Venlafaxine, abilify (aripiprazole)	Abilify changed recently but has been prescribed antidepressants for 8 years	Psychiatrist
			Antipsychotic			

**Table 2:** Clinician participant characteristics.

**Participant identifier**	**Gender**	**Job title and length of time working in the CMHT pathway (at time of interview). Other background information**
Psychiatrist 1	M	Consultant Psychiatrist for over 2 years in this pathway. Qualified as a Consultant a number of years ago and has worked in different mental health teams locally.
Psychiatrist 2	M	Consultant Psychiatrist in this pathway for ~8 months. Previous role was also community based psychiatry. Relatively newly qualified.
Psychiatrist 3	F	Consultant Psychiatrist in this pathway for ~1 year. Qualified as a consultant a number of years ago and has experience of many parts of MH services locally.
Psychiatrist 4	M	Has acted as a Locum Consultant Psychiatrist in the pathway for ~2 months. Previously has worked in many different MH teams and contexts (acute/community) across different parts of the UK.
Psychiatrist 5	F	Consultant Psychiatrist on specialist register. Unknown length of time in pathway, but has worked in the NHS for a number of years.
Psychiatrist 6	F	Consultant psychiatrist in this pathway for 2 years. Number of years experience in other parts of MH service.
Psychiatrist 7	F	Consultant psychiatrist in this pathway for ~3 months. Newly qualified.
CPN 1	F	Worked in pathway for ~2 years. Extensive previous experience of psychiatric nursing.
CPN 2	F	Worked in pathway for over 2 years. Extensive previous experience of community psychiatric nursing.
CPN 3	F	Team leader and community psychiatric nurse. Worked in pathway for over 2 years and extensive previous experience of psychiatric nursing.
CPN 4	F	Worked in pathway for over 2 years. Extensive previous experience of community psychiatric nursing.
CPN5	M	Worked in pathway for over 2 years. Extensive previous experience of community psychiatric nursing.
CPN 6	F	Worked in pathway for ~2 years. Extensive previous experience of community psychiatric nursing.
CPN 7	F	Worked in pathway for over 2 years. Extensive previous experience of community psychiatric nursing.
CPN 8	F	Worked in pathway for over 2 years. Extensive previous experience of community psychiatric nursing.

Three emergent superordinate themes were identified: “Enacting SDM in service users–provider interactions,” “The Therapeutic Relationship as an enabler of SDM,” and “structural challenges to achieving SDM in practice.”

### Enacting SDM in Service Users-Provider Interactions

Four subordinate themes comprise the first broad domain which encapsulates perceived key features of SDM for psychiatric medication management comprising: (1) the importance of SU ownership and control, (2) the dilemma of providing information about adverse effects, (3) a meeting of experts—valuing experiential knowledge, and (4) being ill as a barrier.

#### The Importance of Service User Ownership and Control

Service users, psychiatrists, and nurses viewed SDM in terms of encouraging service users to have increased say over decisions concerning medication, and promoting ownership and self-determination in meetings concerning medication. All three groups strived toward the service user achieving greater self-management skills. Receiving a full explanation of options and gaining detailed information about adverse effects was related to feelings of increased control for service users and referred to specifically by clinicians when describing memorable examples of success. For service users, being able to understand the information about the options were associated with feelings of increased control. However, this was tapered by acknowledging that during periods of crisis increased guidance and less ownership over the decision is possible and the “*sad truth you just need someone to treat you”* (Holly^*^) (see section Being Ill as a barrier). In these more difficult times, having information to take away and revisit was associated with feeling more in control.

*Casey: But I just think if I'd been given that information and going through it yourself and having time to discuss it, you're going to understand. I just think you'd feel like you had more control and, you know, that might reduce stigma, as well as you feeling you can take control of what's going on*.

[^*^ all names are pseudonyms]

*CPN 4: I think she gained an understanding about how medication could be useful and, how, you know, it had its place. But she was. but she was taking control appropriately for when she took it. I felt, yeah, she's got it now, and that left me feeling reassured about her coming off it this time*.

For service users not feeling involved in discussion about options in routine, while not in crises, was associated with feelings of helplessness and lack of control.

*Rosie: The last time that I saw her [the psychiatrist], my medication was increased, and my mood was low but I didn't really know, like I wanted more options and I thought that it would have been better if I had talked it through with her a bit more about increasing the dose and instead she just increased the dose and that' it*.

#### The Dilemma of Providing Information About Adverse Effects

All stakeholder groups stressed the importance of weighing up information and ensuring service users are provided with information about the potential adverse effects of medication options, advocated in standard models of SDM (9). For most service user participants there was a general concern about associated adverse effects and many had previous negative experiences of medication. Many participants referred to not always receiving adequate information. Likewise, most psychiatrists, while stressing the importance of disclosing possible adverse effects, often referred to not doing so due to time constraints and limiting the conversation about what side effects might be important to them. Instead, psychiatrists preferred referring service users to other sources of information, such as leaflets. In addition, while psychiatrists did not think they would deliberately withhold information on adverse effects, some acknowledged that at times, limiting the discussion about adverse effects and possible benefits of the medication was used as a way (consciously or unconsciously) to encourage concordance and avoid possible conflict (28).

*Andrew: One thing is that you are never given enough information about the side effects*.

*Psychiatrist 1: And I'm probably not great about telling people about possible longer term side effects about things and particularly anti psychotics I suppose. I guess there are slightly peculiar circumstances, so if someone's psychotic and has lots of delusions and is fairly wound up in them then a conversation about medication can go a slightly odd way and tend to focus on symptoms that might be otherwise quite secondary*.

*Psychiatrist 2: I think I'd usually say the commonest side effects that other people have mentioned to me about medication, but I usually tell them to look it up on the leaflet I provide, or the internet, because there's no way I can go through all the side effects and I don't know which of the side effects might be important to them*.

#### A Meeting of Experts—Valuing Experiential Knowledge

The vast majority of service users and CPN participants mentioned the importance of service user's experiential knowledge for meaningful sharing of expertise in decisions. The importance of both parties having a say, and equally contributing to the conversation, is seen as integral to SDM. This may be particularly important given acknowledged uncertainty of helpfulness of medication options in this context.

*Linda: Um, I think ideally it should be um, a collaboration between the um, psychiatrist or prescribing doctor and the service user, so there's sort of the knowledge of the different types of medication on the one side and then the SU knows how they are feeling, they know, sort of whether they've got sort of patterns to their moods that sort of certain types of drugs are more able to help with so it's sort of a feedback situation, with both of them contributing*.

Psychiatrists also emphasized the importance of honesty and, at times, disagreement was considered a success (and highlighted this during descriptions of successful meetings). However, CPNs and service users directly expressed the importance of experiential knowledge and a shared dialogue. This aspect was less explicit in the psychiatrists' interviews who instead saw their role as advisors for the evidence base of medication options, which then may subsequently be weighed up by the service user (see also section The importance of service user ownership and control, above).

#### Being Ill as a Barrier

Challenges surrounding being ill or in crisis for service users involvement in medication related decisions were discussed by practitioners and service users. While research highlights that many inpatients remain capable of participating meaningfully during crisis [e.g., (34)] for service users, reinforcing information and increased guidance become of greater importance during crisis than at other times.

*Carl: Um, I think if I'd been in a better place mentally at that time I might have pulled up some questions, um but given how I was at the time, um I don't think I could have done much more because I was looking to be informed by her [the psychiatrist] as much as anything and, you know, that didn't really happen at that point in time*.

Lack of insight, or the SU not accepting that they are mentally ill, was mentioned by both CPNs and psychiatrists as a key challenge to SDM and was associated with changing how information is presented and how medication conversations are constructed. Problems when someone was acutely unwell being framed as “lacking insight” by practitioners is an important issue in that a person's competence to participate is directly challenged by this construction. Service users didn't directly refer to problems of lacking insight, but instead referred to functional problems of poor concentration, memory problems, and distress hindering being able to weigh up and process information about medication or be able express oneself clearly.

*CPN 2: Particularly if someone's very ill and their insight is very poor, and you think, this person really does need to take medication, they're really unwell,… it's important to find out, to support and listen and advise and yet encourage concordance*.

*Psychiatrist 6: If they don't necessarily see it as part of being ill, and then it becomes quite difficult to involve people um on the same level because you have to walk a fine line…. It's not about giving the wrong information but giving information that would lead people to consider perhaps the options more carefully*.

### The Therapeutic Relationship as an Enabler of SDM

Practitioners and service users alike emphasize the importance of achieving a constructive therapeutic alliance and see this as essential. Establishing trust and communicating honestly is seen as an integral aspect of SDM by all participant groups (see section Trust and honesty), yet different conceptualizations about the longer-term, caring, and supportive aspects of relationships emerge between stakeholders (see section Walking the Journey Together and Continuity of Care).

#### Trust and Honesty

Service user participants were aware of the effort required by both parties to establish a deeper relationship and understood that SDM requires honesty on both sides. Several participants referred to not just establishing a general rapport but rather an ongoing effort in building mutual trust. Experiences that denigrated trust were highlighted by service users as particularly damaging for SDM. One participant referred to having to overcome previous issues with trust and having to make a deliberate effort to trust practitioners in order to embark on their own recovery process,

*David: I have to do everything I can to allow myself to get better but if it means I have to trust somebody that I don't know, which is very, very difficult for me to do, then so be it*.

*Carrie: I think the main thing is to be as honest as possible.the honesty and the trust I think as well, and you know you kind of build up a relationship with somebody and you get to trust them*.

When describing memorable positive meetings, psychiatrists referred to establishing rapport and service users feeling able to speak openly and honestly. Some psychiatrists acknowledge that there are potentially differing agendas in conversations concerning medication [and that conflict may emerge when medication is deemed as the best course of action [see (28)]. In this context, service users feeling able to express their views honestly is seen as a particular success. Conversely, issues of false compliance, or service users withholding information about medication was often described as particularly concerning (but a common reality) for many psychiatrists and CPNs.

*Psychiatrist 3: I thought it was really good that she was able to talk frankly about the pros and cons of the medication and she felt she could say she needed this stuff. Yeah, good rapport, trust, a sense that she could say what she really wanted…. [Because] I cannot be absolutely sure that people are being absolutely honest with me and would say. “I don't want to take it.” I mean some of them are going to say, “I got out of there …without losing face” and that's the problem, I don't want people to go away and not take the drug because they can't face me, but they will, some of them.”*

#### Walking the Journey Together and Continuity of Care

Establishing a long-term partnership and supporting people with their personal recovery journeys was seen as integral to the process of SDM for CPNs and SU participants. CPNs referred to the importance of a supportive, long term relationship with SUs. Care and empathy is emphasized reinforcing a deeper level of connection as a way of facilitating a collaborative partnership. Both groups saw this as a continued reflective process, of being held accountable, *pushing forward* and having a belief in one's potential during more difficult times, and celebrating, and reflecting on success over time. For SUs, meaningful involvement was not seen as a series of isolated decisions, but instead, a practitioner seeing and commenting on change over time was connected to feelings of being known and cared about (see Natasha, below).

*CPN 2: So there's a kind of walking the journey together, and sometimes he's pulling back a bit and I'm pushing forward and we were at different paths pulling in different directions. That push - pull stuff, [and] probably the success is about two people building up a mutual respect and real affection for each other [And, for example] thinking “I really care about you.”*

*Natasha: I don't know, I think it's because he [CPN] kept comparing to how I was and how I am. So, you know; look how far you've come, it was all just really positive, rather than “okay, you've taken it, well done.” Yeah, it was real.and he was like; “well done, you know, before you would have stopped and that would have been it but I'm glad you have, you know, I' really proud of you, you've done this, you're doing so well.”*

Overall, psychiatrists didn't emphasize longer term or caring aspects of the therapeutic relationship. Only one psychiatrist (in an example of a successful meeting) described how knowing the person was an important feature of the meeting, and in general, this was seen as not being central to the psychiatrist's role (see also section The changing role of the Psychiatrist, below).

### Structural Challenges to Achieving SDM in Practice

This theme reflects participants' views surrounding the attitudes, structures and cultural challenges of embedding SDM in psychiatric medication management practice. Sub themes include the move toward psychiatrists being seen as performing an expert consultative role (The changing role of the Psychiatrist), construction of distress as a medicalized phenomenon and the associated labeling and stigmas as a barrier to SDM (Medicalization of distress, Labeling, and stigma), and fear of coercion as a barrier (Fear of coercion as a barrier to SDM).

#### The Changing Role of the Psychiatrist

Psychiatrists often discussed the changing role of the psychiatrist toward that of being an expert advisor and performing a consultative role in the medication management process. Five psychiatrists referred to changing roles in recent years, with family doctors (GPs) providing ongoing continuity of care. This connected to the wider trend that longer term care for people occur in the context of primary care and that psychiatrists often only become involved as experts during crisis periods, or in complex cases.

*Psychiatrist 5: Compared to the past, where you would see a patient and would continue to see them for a good length of time, you'd build a relationship and you are overseeing the treatment for a long period of time. From there to now it's moving towardz the GP being the center managing the patients and the consultant psychiatrist providing a sort of consultative model. and there are sort of pros and cons with either. But the current model is one of where you don't see the psychiatrist unless it's um, extremely complex, extremely risky*.

#### Medicalization of Distress, Labeling, and Stigma

Concerns surrounding the dominant discourse of medical understandings of mental health problems and distress emerged as a barrier to collaborative decision making across all three stakeholder groups. Within this, there were differences in conceptualization across participant groups. Service users referred to worries about labeling and stigma associated with a psychiatric diagnosis, and as problematic for SDM. For some participants, diagnosis related to feeling labeled and prejudged impacting feelings of not being valued or listened to in conversations about medication. Ziggy, when describing a memorable negative meeting with his psychiatrist, refers to a pretense of listening by the psychiatrist and feeling ignored: “*And there are some semblances of listening, but it's not really going in because in their mind they've already put a label on me*.” For SUs this theme is also connected to feelings of being spoken down to, of not having a voice, and of a culture of *doctor knows best*. In Natasha's quote below, feelings of being attacked, looked down upon and judged connected to being unable to contribute in a conversation with her psychiatrist.

*Natasha: I felt like I was being attacked and I don't know, it was like I was coming to them for help, it just felt like I was just being attacked and judged and sort of looked down on and it just made me feel really uncomfortable, upset. The fact that I was too scared to say anything, it was, you know, just horrible, it made me feel even worse*.

Stigma was less directly referred to by psychiatrists and CPNs. Instead, for some psychiatrists and CPNs the trend toward an increased medicalization of distress and pathologization of emotions is seen as an emergent and important problem for adopting a holistic approach and open discussions with service users. This was seen as problematic for some CPNs and psychiatrist participants who referred to feeling an increasing pressure to prescribe connected to feeling that service users expect a biomedical explanation and treatment for their mental health problems. Here, passivity and “*wanting to be led*” was seen as a challenge for SDM in mental health.

*Psychiatrist 6: And I think there's quite a push for society to see, um, emotions as abnormal and therefore needing treatment. And I think that's certainly increased in the last couple of years, where I see people who are under distress and find it very difficult to deal with emotions that, um, that are probably, um, a combination of social changes and, um, a kind of breakdown of society's normal coping strategies. So that's my sense*.

#### Fear of Coercion as A Barrier to SDM

The context of mental health services operating within a legal framework and specifically the role of the Mental Health Act (MHA) in removing choice and freedom in the decision making process was acknowledged as a barrier to collaborative decision making by psychiatrist and SU participants. Fear of coercion and the legal context was seen as a particular barrier, hindering honest dialogue, and preventing trust from being established. In one interview a psychiatrist refers to a memorable recent meeting with a service user and reflects that the service user may be withholding information or feeling pressure to take prescribed medication because of his previous experience “*he may be worried that people will cart him off to hospital if he stops taking it*.” Often a general fear of coercion was not necessarily based on direct previous experience of being treated under the MHA, but a general awareness of the legal context.

*Terry: …so I thought to myself; if I get these things going on in my brain, I won't tell a psychiatrist because I don't want to be in hospital*.

*Interviewer: So that's something you've learnt?*

*Terry: Try and be as honest as you can but hold back a little bit because you don't want to sort of end up in hospital when you look different to society*.

Interestingly, fear of coercion was not explicitly mentioned by CPNs, perhaps suggestive of the differing role the CPN performs to the psychiatrist in this pathway.

## Discussion

The findings of this study support broader conceptions of SDM as a longer term process of trial and error, prioritizing honest open dialogue, valuing experiential knowledge, positive risk taking, and viewing psychiatric medication as only one possible choice in a wider personal tool box approach (8, 30, 35).

These findings support other research exploring service users' views of SDM and highlight the enabling role therapeutic relationships play for SDM in mental health (11). Therapeutic relationships and their connection to shared decision making has been relatively unexplored. Research suggests that positive outcomes of therapeutic relationships may be mediated by impacts on increased involvement and SDM (36, 37).

Considerations of how power is enacted in mental health services is critical (38). Insidious forms of power, and perceived labeling, stigma and self-stigma impact SDM directly *via* professional attitudes toward service users. Many SDM professionals view people with SMI as incapable of participating; and service users may internalize associated stigmatizing attitudes, further hindering a person's confidence to express themselves and portray their preferences and values in encounters with professionals (34).

Other forms of power, such as “aesulpian power” (or a power to heal) suggest it is important to recognize that prescribers perform important symbolic and functional roles (39). Yet, for psychiatric medication management, this may lead to an overly medicalized approach, with subsequent reduced emphasis on personal meanings and wider psychological and social understandings of medicines and threatening the ideal of a meeting of experts and of experiential knowledge holding weight encounters (40, 41). A clearer focus on shared risk taking as a way of conceptualizing SDM for such encounters would allow for a diffusion of power and authority and can lead to a meaningful exploration of issues of accountability within the system along with the context of the person's broader life implications (30).

### Implications for Practice

The findings suggest that future interventions to promote SDM in practice need to take a multi-faceted approach, including a focus on changing attitudes amongst mental health practitioners, and empowering service users (12). Recent SDM interventions have advocated for this broader organizational change approach and show early promise, suggesting that attitudes can be effectively changed to support the embedding of SDM in practice (6, 8, 34). However, implementing SDM in mental health settings requires particular attention to the unique defining cultural features of this system. Interventions with a particular emphasis on tackling the insidious effects of labeling and stigma in psychiatry represent an important avenue for future interventions and implementation of SDM in mental health (34). The importance of co-production in the development and implementation of interventions to embed SDM in practice, may be particularly important in this regard (20). In addition, promoting continuity of care and longer term relationships is an important practical implication of the findings. The findings suggest that the role of the CPN for collaborative psychiatric medication management practices may be particularly important. In the UK, an increasing fragmentation of services, and increased emphasis on time-limited focused provision leads to increasing concerns about the impact service design has on health outcomes *via* reduced continuity of care, both within and across different pathways (42, 43).

### Strengths and Limitations of This Study

Presentation of themes across three stakeholder groups (CPNs, service users and psychiatrists) allowed for discussion of complexities and areas of convergence and divergence in themes between stakeholder groups. Arguably incorporating multiple stakeholder groups also allows for a more sophisticated construction. The co-produced elements of the research process, and the inclusion of collaborative analysis phases enabled through active involvement from the SU advisory group during the analysis phases strengthened the credibility and quality of the results. However, it may be that by highlighting differences between groups, an artificial portrayal of homogeneity in views within stakeholder groups is presented, portraying a crude distinction that, for example, psychiatrists have one position on this topic, and CPNs or service users another. This was not the explicit intention and hopefully we were able to portray the complexity of the themes and present both inter as well as intra group differences in discourse.

The fact that participants were all recruited from CMHT in one locality, should be considered in relation to relevance of findings to other service users and practitioner populations. It is also possible that selection bias was present in the recruitment of service users participants within the pathway (this issue doesn't apply to interviews with professionals in that all but one participated). It may be that those service users with existing good relationships were approached for participation by gatekeepers (mental health professionals in the CMHT). Also, people with strong views or memorable experiences on the topic may have been more interested in participating. As such, future research should seek to recruit a more inclusive and varied service user sample and involve a larger more representative sample.

### Concluding Remarks

This is among the first studies to explore *both* practitioner and service user perceptions of SDM for psychiatric medication management. This study's importance is also reflected in the inclusion of CPN's views (an under researched group) alongside service user and psychiatrist views about SDM for medication management is an important contribution. Currently there is insufficient research examining wider cultural and structural enablers and barriers for successful implementation in everyday psychiatric practice. These results offer a detailed contextualized account of how medication decisions are made and highlight that SDM is a long-term endeavor embedded within therapeutic partnerships. Stakeholder differences in views of SDM reflect a complexity of relations which point to wider system and cultural challenges at different stages of the SDM process. The study provides actionable insights which may help improve SDM practices and improve the quality of psychiatric care.

## Data Availability Statement

De-identified data that support the findings of this study are available from the corresponding author upon reasonable request.

## Ethics Statement

The studies involving human participants were reviewed and approved by Hertfordshire Research Ethics Committee, NHS. The patients/participants provided their written informed consent to participate in this study.

## Author Contributions

EK was lead researcher for this paper, coordinating the data collection and analysis, and final drafting of findings. SR contributed to the analysis of findings and drafting the paper.NM contributed to the analysis of findings and drafting of findings. YZ-I contributed to the wider discussion of literature and links to other literature in the field. All authors contributed to the article and approved the submitted version.

## Conflict of Interest

The authors declare that the research was conducted in the absence of any commercial or financial relationships that could be construed as a potential conflict of interest.

## Publisher's Note

All claims expressed in this article are solely those of the authors and do not necessarily represent those of their affiliated organizations, or those of the publisher, the editors and the reviewers. Any product that may be evaluated in this article, or claim that may be made by its manufacturer, is not guaranteed or endorsed by the publisher.
