# Corrigendum: Barriers and Enablers to Shared Decision Making in Psychiatric Medication Management: A Qualitative Investigation of Clinician and Service Users' Views

**DOI:** 10.3389/fpsyt.2021.789916

**Published:** 2022-03-14

**Authors:** Emma Kaminskiy, Yaara Zisman-Ilani, Nicola Morant, Shulamit Ramon

**Affiliations:** ^1^School of Psychology and Sports Science, Anglia Ruskin University, Cambridge, United Kingdom; ^2^Department of Social and Behavioral Sciences, College of Public Health, Temple University, Philadelphia, PA, United States; ^3^Department of Clinical, Educational and Health Psychology, Division of Psychology and Language Sciences, University College London, London, United Kingdom; ^4^Division of Psychiatry, Faculty of Brain Sciences, University College London, London, United Kingdom; ^5^School of Health and Social Work, University of Hertfordshire, Hatfield, United Kingdom

**Keywords:** shared decision making, barriers, facilitators, co-production, medication, psychiatry, coercion, stigma

Nicola Morant was not included as an author in the published article. The author list and affiliations have been corrected and the new Author Contributions Statement appears below.

In the published article, there was an error. The acknowledgments statement was missing. The statement appears below.

## Author Contributions

EK was lead researcher for this paper, coordinating the data collection and analysis, and final drafting of findings. SR contributed to the analysis of findings and drafting the paper. NM contributed to the analysis of findings and drafting of findings. YZ-I contributed to the wider discussion of literature and links to other literature in the field. All authors contributed to the article and approved the submitted version.

## Publisher's Note

All claims expressed in this article are solely those of the authors and do not necessarily represent those of their affiliated organizations, or those of the publisher, the editors and the reviewers. Any product that may be evaluated in this article, or claim that may be made by its manufacturer, is not guaranteed or endorsed by the publisher.

